# Human FcRn Tissue Expression Profile and Half-Life in PBMCs

**DOI:** 10.3390/biom9080373

**Published:** 2019-08-15

**Authors:** Yao-Yun Fan, Vahid Farrokhi, Teresa Caiazzo, Mengmeng Wang, Denise M. O’Hara, Hendrik Neubert

**Affiliations:** Biomedicine Design, Pfizer Worldwide Research & Development, Andover, MA 01810, USA

**Keywords:** antibody, human FcRn, half-life, IA-LC-HRMS, tissue-based target quantification, PBPK

## Abstract

System-wide quantitative characterization of human neonatal Fc receptor (FcRn) properties is critical for understanding and predicting human PK (pharmacokinetics) as well as the distribution of mAbs and Fc-fusion proteins using PBPK (physiologically-based pharmacokinetic) modeling. To this end, tissue-specific FcRn expression and half-life are important model inputs. Herein, human FcRn tissue expression was measured by peptide immunoaffinity chromatography coupled with high-resolution mass spectrometry. FcRn concentrations across 14 human tissues ranged from low to 230 pmol per gram of tissue. Furthermore, the FcRn half-life was determined to be 11.1 h from a human stable isotope labelled leucine pulse labeling experiment. The spatial and temporal quantitative human FcRn data now promise to enable a refined PBPK model with improved accuracy of human PK predictions for Fc-containing biotherapeutics.

## 1. Introduction

Neonatal Fc receptor (FcRn) plays a key role in extending the serum half-life of therapeutic antibodies [1,2]. Understanding the distribution and expression dynamics of FcRn in target and surrounding tissues is anticipated to aid with establishing a more informed and improved physiologically-based pharmacokinetic (PBPK) model for PK prediction of Fc-based biotherapeutics [3,4,5]. Previously, FcRn detection either via its mRNA or at the protein level using immunoblotting has been reported from selected mouse and human tissues [6,7,8]. In general, FcRn tissue expression was shown to correlate with the endothelial and epithelial cell number in the tissue [9,10]. The online peptide immunoaffinity enrichment approach has recently enabled sensitive mass spectrometry measurements and quantitation of human FcRn in transgenic mouse tissues [11]. However, the comprehensive quantification of FcRn expression in human tissues using a mass spectrometry approach has not yet been reported.

Furthermore, stable isotope-labeled (SIL) leucine pulse and pulse-chase labeling in human subjects followed by protein immunoaffinity and targeted mass spectrometry has been applied for measurement of turnover of target proteins [12,13,14,15]. In this study, we measured the FcRn expression profile in human tissues as well as FcRn half-life in peripheral blood mononuclear cells (PBMCs) collected from a U-^13^C-leucine pulse labeling study in normal human healthy volunteers.

## 2. Materials and Methods 

### 2.1. Rabbit Polyclonal anti Human FcRn Antibody Generation

New Zealand white rabbits were injected subcutaneously (SC) on days 0, 28 and 70 or intramuscularly (IM) on days 14 and 42 with 100 μg of recombinant human FcRn (rhFcRn; both alpha chain and β2microglobulin, Arvys Proteins) conjugated to keyhole limpet hemocyanin (KLH) at a molar coupling ratio of 0.05:1 (KLH:rhFcRn). All procedures performed on animals were in accordance with regulations and established guidelines and were reviewed and approved by an Institutional Animal Care and Use Committee (Animal welfare assurance number: A3997-01; AAALAC accreditation number: 001032; USDA research license number: 23-R-0122). rhFcRn-KLH was formulated and administered with complete Freund’s adjuvant initially followed by incomplete Freund’s adjuvant at 14-day intervals. Sera were collected, screened for reactivity to rhFcRn and subsequently pooled based upon antibody titers. 

### 2.2. Ligand Affinity Purification of Rabbit Polyclonal FcRn Antibody

Pooled rabbit antisera were diluted 1:1 with PBS (20 mL *v*/*v*) and ligand affinity purified by ÄKTA™ FPLC employing a HiTrap NHS 1 mL column (GE Healthcare, Princeton, NJ, USA) coupled with rhFcRn ligand. Fractions containing rabbit anti-hFcRn antibodies were eluted with 100 mM glycine-HCl, pH 2.5 and neutralized with 1M Tris, pH 9.0 buffer. Fractions were combined based on their A280 measurement and dialyzed into PBS in a 10K MWCO slide-a-lyzer (Thermo Fisher Scientific, Waltham, MA, USA) vessel for 18–24 h at 4 °C.

### 2.3. Biotinylation of Ligand Affinity Purified Rabbit Polyclonal FcRn Antibody

Ligand affinity purified antibodies were conjugated to EZ-Link™Sulfo-N-hydroxysuccinimide (NHS)-LC biotin (Thermo Fisher Scientific, Waltham, MA, USA) at a molar coupling ratio of 20:1 (biotin:antibody) for 2 h at ambient temperature. After quenching with 1M Tris, pH 9.0, the reaction was buffer exchanged into PBS as described above followed by storage at −70 °C or below.

### 2.4. FcRn Tissue Concentration Measurements

Based on the established methodology of human FcRn analysis in Tg32 transgenic mice [11], human tissue-processing workflow was similar, with the addition of using dissection scissors to cut tissues in lysis buffer in eppendorf tubes prior to adding stainless beads for homogenization. In brief, tissues were lysed with TER-I buffer (FNN0071, Thermo Fisher Scientific, Waltham, MA, USA) with protease inhibitor (78430, Thermo Fisher Scientific) into 50 mg/mL. Frozen human normal PBMCs were purchased from BioIVT (Westbury, NY, USA). Frozen cell pellets of 50 million cells were reconstituted and lysed with 500 µL of TER-I buffer. A 400 µL aliquot of tissue lysate or cell lysate was precipitated in cold acetone (1:4, *v*/*v*) at −20 °C for 1 h and centrifuged at 4 °C, 10,000× *g* for 15 min. Protein pellets were washed with cold acetone, dried, and solubilized with 250 µg of TPCK-trypsin (T1426, Sigma, St. Louis, MO, USA). Five-hundred femtomoles of stable isotope-labeled (SIL) peptides were spiked into 40 µL of solubilized lysate. Samples were reduced with the addition of 10 µL of 70 mM dithiothreitol (DTT) for 45 min at 60 °C and alkylated with the addition of 10 μL of 140 mM iodoacetamide (IAA) at room temperature in the dark for 45 min. Details of LC and MS settings have been described previously [11]. In short, peptides from tissue lysates were diluted with 1 M urea in 25 mM ammonium formate (1:9, *v*/*v*), followed by loading 100 µg onto FcRn anti-peptide antibody column, washed with 500 mM ammonium formate, eluted with 0.4% trifluoroacetic acid onto PepMap 300 C18 trap cartridge (5 mm × 0.3 mm, 5 µm, 300 Å) of Ultimate 3000 (Thermo Fisher Scientific, Waltham, MA, USA) nano LC system. Peptides were chromatographically separated by PepMap C18 analytical column (EASY-Spray™, PepMap 3 µm, 100 Å, 75 µm i.d. × 15 cm). Analyte peptides were analyzed by Q Exactive using high-resolution tSIM at 140,000 fwhm at *m*/*z* = 200. Data processing and quantification was done with Pinpoint software, version 2.0 (Thermo Fisher Scientific, Waltham, MA, USA), where three precursor isotopes were summed for quantification.

### 2.5. Heavy Leucine Pulse Labeling in Human Volunteers 

The stable isotope-labeled leucine pulse labeling was performed with infusion of U-^13^C-leucine in healthy human volunteers. Clinical characteristics of the two subjects in this study are listed in Supplementary Table S1. PBMC samples from two subjects were collected longitudinally for the study. Clinical study was performed at the Profil Institute for Clinical Research (Chula Vista, CA, USA) and was approved by an institutional review board with informed consent from all subjects. Subjects were admitted on Day 1 at 9 AM and had breakfast. Standard (ad libitum) lunch was provided at approximately 12 PM and ad-libitum dinner was provided at approximately 5 PM on Day 1. An intravenous (IV) line was placed on one arm for blood drawing and another IV line in the contralateral arm for the leucine infusion was placed before the start of the leucine infusion. Blood was collected before the start of the leucine infusion in Day 1. Primed intravenous infusion of 242 mg of labeled leucine was administered over 5 min at approximately 6 PM on Day 1 and that was followed by a 145.2 mg/h continuous infusion for 36 h to achieve plasma steady-state leucine enrichments levels above 10%. During the leucine infusion on Day 2, low-protein meals consisting of ≤ 40 g protein per day were provided to the subjects. Blood samples were collected before and during the leucine infusion on Day 1, Day 2 and Day 3 at time points that are presented in Supplementary Table S1. At each time point, blood was collected in 1 × 8.5 mL BD™ P100 collection tubes (#366448, BD Biosciences, Franklin Lakes, NJ, USA) for plasma preparation, and a separate blood sample was collected in 1 × 8 mL Cell Preparation tubes (#362761, BD Biosciences, Franklin Lakes, NJ, USA) for PBMCs and other blood cell product collections. In a third vial, at every other time point, blood was collected in 1 × 2 mL K_2_EDTA collection tubes (#367841, BD Biosciences, Franklin Lakes, NJ, USA) for the measurement of heavy leucine enrichment in plasma. PBMCs were prepared following the vendors protocol. Samples (plasma and cells) were frozen and stored at −80 °C until analysis.

### 2.6. FcRn Half-Life Measurement

Collected PBMCs (about 10 million cells) were thawed on ice and lysed in 200 µL of TER-I lysis buffer with protease inhibitor with stainless-steel beads using a Bullet Blender (Next Advance, Troy, NY, USA) at 4 °C on speed setting 4 for 5 min. The lysates were centrifuged at 13,000× *g* at 4 °C for 15 min and the supernatants were transferred into a LoBind 96-well plate (Eppendorf, New York, NY, USA). Each lysate was diluted with the addition of 200 µL calcium and magnesium free phosphate-buffered saline with 0.05% tween 20 (CMF-PBST), pH 7.2, and 1µg of biotinylated anti-FcRn antibody was added to each sample. Samples were incubated overnight at 4 °C with gentle mixing. Twenty mictroliters of pre-washed streptavidin T1 MyOne Dynabeads (Invitrogen, Carlsbad, CA, USA) were added to each sample and incubated on a mixer at room temperature for 45 min. Automated bead processing steps were described previously [12,16,17]. Briefly, captured proteins were eluted from washed beads with the addition of 140 µL of 30 mM HCl and neutralized with mixing with 30 µL of 1 M Tris HCl pH 8. Samples were reduced and alkylated as mentioned above. One microgram of trypsin/Lys C mixture (Promega, Madison, WI, USA) was added to each sample and incubated overnight at 37 °C on a thermomixer for digestion completion.

From the digested protein mixtures, 50 µL was injected into a Ultimate 3000 (Thermo Fisher Scientific, Waltham, MA, USA) nano LC system with a PepMap 300 C18 trap cartridge (5 mm × 0.3 mm, 5 µm, 300Å) in 0.5% trifluoroacetic acid solvent at a flow rate of 100 µL/min for 3 min. Peptides were eluted into and separated in an analytical column (EASY-Spray™, PepMap 3 µm, 100 Å, 75 µm i.d. × 15 cm), applying a 10 min gradient from 2% to 45% organic solvent (98% ACN, 0.1% formic acid) with a total run time of 16 min, and analyzed by TSQ-Quantiva™ triple quadrupole mass spectrometer (Thermo Fisher Scientific, Waltham, MA, USA). Details of MS settings have been described [12]. 

## 3. Results and Discussions

**Figure 1:**
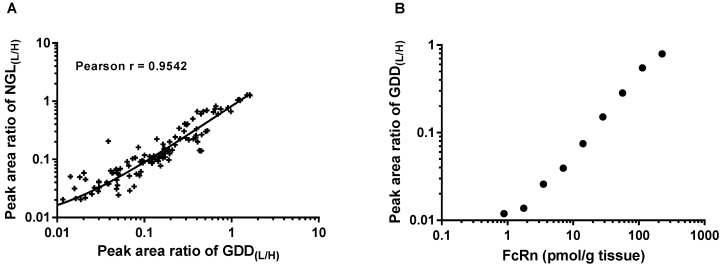
FcRn measurement in human tissues. (**A**) Correlation of peak area ratio from measured FcRn peptide (Light, L) to SIL internal standard (Heavy, H) of human tissue samples between quantification (GDD) and confirmatory (NGL) peptides, with a high Pearson r score of 0.95. (**B**) Quantification range of hFcRn in tissue was 0.89–226.35 pmol/g tissue using the peak area ratio of the GDD peptide. Shown is the average of a duplicate calibration curve.

For human tissue-specific FcRn quantification, multiple FcRn surrogate peptides were directly adapted from the established methodology previously reported for the quantification of human FcRn in Tg32 transgenic mice [11]. The peptide sequence GDDTGVLLPTPGEAQDADLK (GDD) was selected as the quantification peptide and is located in the cytosolic domain of human FcRn. The peptide QGTWGGDWPEALAISQR previously used for the human FcRn measurement in Tg32 mouse tissue was not suitable for quantitative analysis in human tissues, as matrix background signals interfered with sensitive analysis. Subsequently, the peptide NGLAAGTGQGDFGPNSDGSFHASSSLTVK (NGL), which is located in the extracellular domain of human FcRn, was selected as the confirmatory peptide for FcRn quantification in human tissues. The peak area ratios from light and corresponding heavy stable isotope-labelled peptide standards (L/H) were determined for GDD and NGL peptides from all human tissue measurements in this study. There was generally good agreement with a high Pearson r score of 0.95, as shown in Figure 1A, providing confidence that the human FcRn quantification was robust and confirming the integrity of the receptor by concurrent monitoring of peptides from both the extracellular and cytosolic domain. Calibration standards were recombinant full-length human FcRn protein spiked into control mouse liver tissue lysates, and GDD peptide was selected as a quantification peptide due to better sensitivity. The quantification range was 0.89−226.35 pmol/g tissue (Figure 1B). The selected analyte peptides are proteotypic to human FcRn and are not conserved in mouse FcRn or elsewhere in the mouse proteome. 

**Figure 2:**
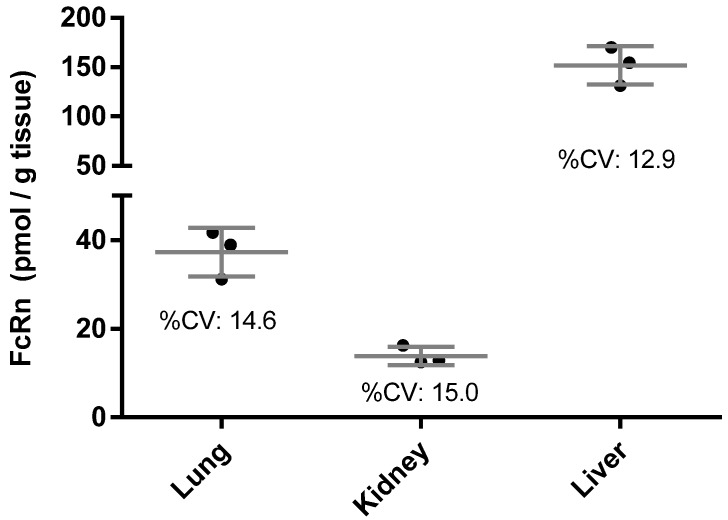
Reproducibility of FcRn measurement in normal human liver, lung and kidney tissues from independently prepared lysates from dissected tissue portions of the same tissue sample.

To ensure assay translation from human FcRn Tg32 transgenic mice to humans and to evaluate the heterogeneity of FcRn expression in human tissues, the intra-tissue variability of human FcRn concentrations measured from various dissected portions from the same human tissue piece was evaluated. Initially, lung, liver, and kidney tissue were used for this purpose., The intra-tissue variability was found to have a 15% coefficient of variation (CV) or less (Figure 2). Subsequently, an assay qualification was conducted with three technical replicates prepared in each experimental set to assess the intra-batch assay performance. Three independent preparations were carried out to assess the inter-batch assay precision and relative accuracy in human tissue matrices. Each set of experiments was composed of human tissue samples of lung and kidney from (I) endogenous human tissue lysate diluted with control wild type mouse tissue lysate; (II) endogenous human tissue lysate and (III) endogenous human tissue lysate spiked with rhFcRn protein. An admix of tissue samples as well as the diluted sample with control mouse tissue lysate were also included in the qualification to demonstrate the robustness of the assay in the presence of various tissue matrices. Table 1 shows the summary statistics from the FcRn assay qualification in human tissues. The intra and inter assay variability of measurement, i.e., precision, was found to be less than 15% CV in all cases. The intra and inter assay relative accuracy was usually no less than 75 to 80%. 

**Figure 3:**
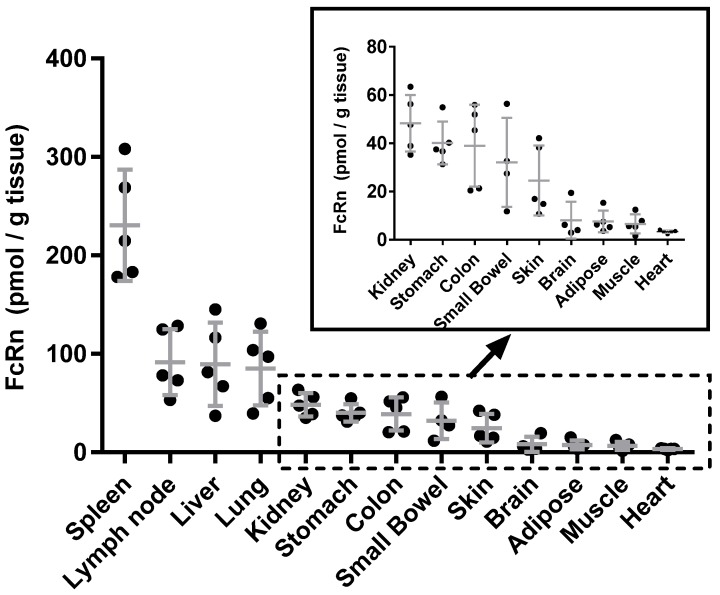
Human FcRn tissue expression profile in humans and a zoomed-in profile for the lower expressing tissues of the individual tissue samples (dots). Error bars represent standard deviations in each tissue type.

Frozen human tissue samples of adipose, brain, colon, heart, kidney, liver, lung, lymph nodes, muscle, pancreas, skin, small bowel, spleen, and stomach were obtained from five independent subjects. Samples were sourced either from normal tissue adjacent to tumors being surgically removed, or from normal post-mortem tissues not directly associated with the cause of death. Each tissue sample was obtained from independent male individuals, aged between 20 and 50 years. The tissue samples are from institutional review board approved collections from multiple sources with informed consent. More detailed sample information is provided in Supplementary Table S2. The measured human FcRn tissue expression is shown in Figure 3, where the insert shows an expanded view of FcRn expression from lower FcRn-expressing tissues. Average tissue concentrations are provided in Table 2. Spleen tissue showed the highest FcRn expression normalized by tissue weight followed by lymph node, liver and lung. Pancreas was found to have the lowest FcRn expression, which was still detectable, but below the established lower limit of quantification, i.e., below 0.89 pmol/g tissue, and therefore not included in the data summary. Peripheral blood mononuclear cells (PBMCs) from three healthy donors were also subjected to FcRn measurement. FcRn concentrations were measured as 0.25, 0.23, and 0.19 pmol per million cells, averaging 0.22 pmol per million cells, which offers quantitative correlation with previously-reported FcRn expression in monocytes and hematopoietic cells [18,19].

The FcRn expression profile in human tissues shown herein is the first reported quantitative data set determined using a qualified mass spectrometry-based method. As demonstrated, the methodology offers robust quantification of FcRn to provide confident FcRn concentration assignments. The highest FcRn expressions on a tissue weight normalized basis were observed in the spleen and lymph node of human and Tg32 transgenic mice [20]. This is consistent with the intensity ranking of tissue staining from mouse models [21], and agrees well with the role of FcRn in regulating IgG (immunoglobulin G) serum half-life. Our report shows quantitative support to previously reported FcRn characterization studies in human liver and kidney [8,22,23]. The rank order of FcRn protein expression in the panel of human tissues is similar to hFcRn inTg32 homozygous mouse [20], with the exception of skin and heart tissues that have higher FcRn expression in Tg32 mouse. This data also supports the utility of the hFcRn Tg32 mouse as a preclinical model to predict human clearance for monoclonal antibodies [24]. 

**Figure 4:**
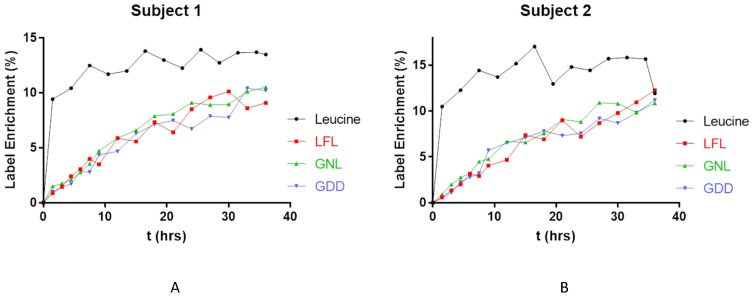
Levels of labeled leucine enrichment in plasma of Subject 1 (**A**) and Subject 2 (**B**) were shown by the black line. Tracer leucine incorporation into FcRn was monitored by three FcRn peptides, LFL (red line), GNL (green line), and GDD (blue line).

For FcRn turnover measurements in human PBMCs, a pulse labeling experiment was performed by the infusion of stable isotope-labeled leucine (U-^13^C-leucine) to two healthy human volunteers as previously described [13]. PBMC samples were collected longitudinally over a 36 h period including at time 0, i.e., before administration of the labeled leucine. A protein immunoaffinity enrichment method was required that would not consume the entire sample as the supernatant of each pulse labelling sample can be used for subsequent turnover analysis of other proteins of interest [12]. Cells were lysed and FcRn was immunoprecipitated using an anti-human FcRn antibody. The FcRn-enriched sample was then digested prior to the analysis by nanoflow LC-MS/MS. The ratio of newly synthesized FcRn peptide (heavy) to total peptide (H/Total) was measured at each time point to assess the incorporation of heavy leucine into the FcRn peptides. Three leucine-containing peptides from human FcRn were selected for the label-enrichment measurements. Two peptides of LFLEAFK (LFL) and GNLEWK (GNL) were from the extracellular domain and peptide GDDTGVLLPTPGEAQDADLK (GDD), which was used for FcRn quantification as described above and was from the cytoplasmic domain of FcRn. For peptides that contained more than one leucine, ion transitions were selected in such a way that peptide isotopologues with only one heavy leucine incorporated were measured. For example, in the LFL peptide, ion transition for y5 fragment, 434.25 [M^2+^] > 607.34 [M^1+^] in the light channel, and ion transition of the same fragment ion, 437.26 [M^2+^] > 613.37 [M^1+^] was measured in the heavy channel. Similar considerations were taken for GDD peptide measurements. With this transition arrangement as shown in Table 3, in peptides with more than one leucine, only one of the leucine residues was considered for the labeled peptide measurements to avoid accounting for double-label incorporation. Also, peptides that were labeled on more than one leucine residue were ignored due to negligible abundance. This was important to hold the first order kinetics assumptions applicable to the measurement results. Data showed that an initial rapid ramp of the tracer levels in plasma was achieved with bolus infusion of labeled leucine at time 0. The levels of labeled leucine in plasma reached about 15% or slightly below and were maintained by continuous IV infusion, as shown in Figure 4. 

The incorporation of tracer leucine into three measured FcRn peptides started immediately after the time zero, and labeled peptides were already observed in the first collected sample after time 0, i.e., at 1.5 h. However, the maximum label incorporation of about 10% was reached at the end of the 36 h infusion. Label-enrichment profiles follow a similar trend in all three peptides with good agreement confirming that the peptides originate from the same intact protein, i.e., similar data can be obtained from leucine-containing peptides from cytosolic and extracellular regions of FcRn. An averaged enrichment profile was prepared using mean values at each time point and was used for data fitting and half-life calculation for each subject. Protein synthesis kinetics information was extracted from fitting both the leucine precursor data and peptide data using SAAM II software [12,25]. When applying a first order kinetics relationship between leucine (precursor) and the peptide (product), the synthesis rate constant (k_syn_) was derived and an average half-life of 11.1 h was calculated for FcRn in PBMCs of two healthy volunteers, as shown in Table 4. FcRn turnover kinetics was previously measured by ^35^S-methionine labeling in EA.hy926 cells, an endothelial cell line, which showed a similar, but slightly longer half-life of 15.6 h compared to the present measurements in human PBMCs [6].

One important assumption in this study was that there is immediate access to the leucine precursor pool by PBMCs. This assumption allows the use of the plasma leucine enrichment data for the protein kinetics measurements in PBMCs. Previous studies have shown that with stable isotope-labeled amino acid pulse-chase methods, the half-life of soluble proteins with very fast turnover rate << 0.5 h can be measured in serum [14]. This implies that the labelled amino acid distributes rapidly and is readily available to the cellular protein synthesis apparatus upon IV administration.

In this report, we presented spatial (tissue expression) and temporal (turnover) measurements of human FcRn, two critical physiological parameters for pharmacokinetics modelling of mAb and Fc-fusion proteins. With FcRn expression at the individual tissue level and FcRn turnover incorporated into PBPK modeling, an improved accuracy of the models is expected to enable a better prediction of the pharmacokinetics of Fc-containing therapeutics. Furthermore, this data is also expected to assist in the development of PBPK models for predicting the effects of anti-FcRn therapy, for example on the disposition of endogenous IgG in humans. 

## Figures and Tables

**Table 1:** Summary statistics of human FcRn tissue assay qualification.

Tissue	Dilution/Spike	Precision (%CV) **				Relative Accuracy % ***			
		Intra-Batch 1	Intra-Batch 2	Intra-Batch 3	Inter-Batch	Intra-Batch 1	Intra-Batch 2	Intra-Batch 3	Inter-Batch
Human Lung	* Dilute 4 fold	1.0	6.5	13.1	6.9	−2.3	23.7	15.3	12.2
	* Dilute 2 fold	4.5	10.0	5.6	6.7	−5.7	−8.2	−10.7	−8.2
	Endogenous	5.9	7.0	6.2	6.3	n/a	n/a	n/a	n/a
	Spike 100 ng/mL rhFcRn	10.0	10.8	7.6	9.5	22.2	11.8	11.9	15.3
Human Kidney	* Dilute 2 fold	9.5	5.7	6.9	7.4	23.2	6.5	2.8	10.8
	Endogenous	11.0	10.9	12.2	11.4	n/a	n/a	n/a	n/a
	Spike 100 ng/mL rhFcRn	10.6	6.8	8.6	8.7	12.8	−7.8	−8.4	−1.1
Admix	* Dilute 2 fold (hLung + hKidney)	6.4	12.9	13.0	10.7	3.4	−13.7	−11.5	−7.3
	Endogenous (hLung + hKindey)	10.3	6.7	6.6	7.9	−10.8	−24.9	−19.9	−18.5

* Use the same tissue from control mouse to make tissue dilutions. ** Intra-batch precision (%CV) was calculated as the ratio percentage of the standard deviation to the mean of each intra-batch triplicate measurement, whereas the inter-batch %CV was calculated as the ratio percentage of the average standard deviation to the grand mean from all batches. *** Relative accuracy (%) was calculated as the percent error between measurement and nominal value.

**Table 2:** Human FcRn tissue expression (average ± standard deviation).

Tissue Type	FcRn Expression (pmol/g Tissue) (Individual Measurement Listed)
Spleen	230.7 ± 56.4 (178.3; 183.2; 308.2; 214.9; 268.8)
Lymph node	91.7 ± 33.4 (124.9; 78.2; 128.7; 73.2; 53.3)
Liver	89.5 ± 42.3 (67.3; 37.0; 81.3; 116.6; 145.2)
Lung	85.2 ± 37.3 (55.0; 130.7; 97.1; 103.8; 39.5)
Kidney	48.3 ± 11.7 (35.3; 38.9; 47.6; 56.2; 63.5)
Stomach	40.1 ± 8.9 (40.2; 31.3; 36.7; 37.4; 54.9)
Colon	39.0 ± 17.0 (20.5; 45.4; 21.3; 55.9; 51.9)
Small bowel	32.1 ± 18.5 (56.3; 11.8; 27.5; 32.6; bloq)
Skin	24.6 ± 14.5 (42.1; 16.9; 38.2; 10.7; 14.8)
Brain	8.1 ± 7.7 (19.4; 6.2; 4.0; 2.9; bloq)
Adipose	7.6 ± 4.5 (5.3; 15.3; 6.3; 3.6; 7.5)
Skeletal muscle	6.5 ± 4.0 (12.4; 5.3; 7.8; 1.6; 5.6)
Heart	3.3 ± 0.7 (4.2; 3.6; 2.3; 3.3; 3.4)
PBMCs	0.22 ± 0.03 (pmol/million cells) (0.25; 0.23; 0.19)

Bloq: below limit of quantification (below 0.89 pmol/g tissue).

**Table 3:** Light and heavy peptide ion transitions used for FcRn turnover measurement.

Peptide	Light		Heavy	
	Q1 *m/z*	Q3 *m/z*	Q1 *m/z*	Q3 *m/z*
LFL	434.25 [M^2+^]	607.34 [M^1+^] y5	437.26 [M^2+^]	613.36 [M^1+^] y5
GDD	1006.49 [M^2+^]	1241.60 [M^1+^] y12, 1043.50 [M^1+^] y10	1009.50 [M^2+^]	1247.62 [M^1+^] y12, 1049.52 [M^1+^] y10
GNL	373.69 [M^2+^]	575.31 [M^1+^] y4, 462.23 [M^1+^] y3	376.70 [M^2+^]	581.33 [M^1+^] y4, 462.23 [M^1+^] y3

**Table 4:** The measured half-life of FcRn in PBMCs from two human volunteers.

Human Subject	k_syn_	Half-Life (t_½_) in h.
Subject 1	0.065	10.6
Subject 2	0.060	11.6

## Supplementary Materials

The supplementary materials are available online at https://www.mdpi.com/2218-273X/9/8/373/s1.
