# Supplementary material for: Human FcRn Tissue Expression Profile and Half-Life in PBMCs
Source: Biomolecules. 2019 Aug 15;9(8):373. doi: 10.3390/biom9080373 (PMC6722552; doi:10.3390/biom9080373)
Supplement: Supplementary file 1 [file biomolecules-09-00373-s001.pdf]

Supplementary Table 1

Clinical characteristics of human healthy volunteers using for FcRn turnover assessment.

**Subject ID**	**1**	**2**
**Age (yrs)**	26	37
**Gender**	Male	Male
**Height (cm)**	186	169
**Weight (kg)**	89.5	81.7
**BMI (kg/m**	25.9	28.6
**Race**	African American	Caucasian
**Selected time points of blood collection (Hr)**	0, 1.5, 3, 4.5, 6, 7.5, 9, 12, 15, 18, 21, 24, 27, 30, 33, 36	

Supplementary Table 2

Human tissue sample used for FcRn concentration measurement.

Number	Tissue type	Age (All male)	Tissue Detail	Tissue sample freeze time interval (Hour ' Minutes'')	Tissue source	Sample ID	Note
1	Adipose	30	PM	1'	ILSBIO	ILSPM1848H-DS1	MVA
2	Adipose	50	PM	3'	ILSBIO	ILS1074BE02	MVA
3	Adipose	25	PM	2'	ILSBIO	ILSPM5025X5	MVA
4	Adipose	24	PM	2'	ILSBIO	ILSPM5032X01	MVA
5	Adipose	40	PM	2'	ILSBIO	ILSB1012D02	MVA
6	Brain	20	PM		ILSBio	ILSC013	
7	Brain	28	PM	2'45"	ILSBIO	ILS10168B02-DS2	MVA
8	Brain	21	PM	4'30"	ILSBIO	ILS11595B2	MVA
9	Brain	44	PM	3'30"	ILSBIO	ILS11584B1	MVA
10	Brain	37	PM; adjacent normal	2'45"	Proteogenex	53332A	Toxic encephalopathy polyneuropathy
11	Colon	48	NAT		Cleveland Clinic	107C055Ca	Surgery
12	Colon	49	NAT		Cleveland Clinic	301C203Cc	Surgery
13	Colon	37	NAT		Cleveland Clinic	209C084Cc	Surgery
14	Colon	50	NAT		Cleveland Clinic	212C056Cc	Surgery
15	Colon	44	NAT		Cleveland Clinic	212C099Cc	Surgery
16	Heart	31	Adjacent normal		Asterand	6160A	
17	Heart	23	PM	2'25"	ILSBIO	ILS10401G04-DS1	MVA
18	Heart	50	PM	3'	ILSBIO	ILS1074BF03-DS1	MVA
19	Heart	25	PM	4'	ILSBIO	ILS20280IB-J1	MVA
20	Heart	40	PM	2'	ILSBIO	ILSPM5026X16	MVA
21	Kidney	50	NAT	1'	Proteogenex	05463T1	Surgery
22	Kidney	43	NAT	1'	Proteogenex	05475T1	Surgery
23	Kidney	46	NAT	1'	Proteogenex	05975T1	Surgery
24	Kidney	29	NAT		Tissue solutions	05928T1	Surgery
25	Kidney	46	NAT		Tissue solutions	05927T1	Surgery
26	Liver	43	NAT	1'	Proteogenex	09259T1	Surgery
27	Liver	37	NAT	1'	Proteogenex	09268T1	Surgery
28	Liver	44	PM; adjacent normal	5'	Proteogenex	09309A	Chronic alcoholism
29	Liver	48	NAT	1'	Proteogenex	09426T1	Surgery
30	Liver	44	NAT	1'	Proteogenex	09517T1	Surgery
31	Lung	49	NAT		Cornell	01-0485 4-2	Surgery
32	Lung	46	NAT		Cornell	01-0542 4-2	Surgery
33	Lung	43	NAT	1'	Proteogenex	041953B1	Surgery
34	Lung	46	NAT	1'	Proteogenex	042037T1	Surgery
35	Lung	21	NAT	1'	Proteogenex	042291T1	Surgery
36	Lymph Node	46	NAT	12"	ILSBIO	ILS30036M1	Colon cancer, Mesenteric lymph node

37	Lymph Node	41	NAT	11"	ILSBIO	ILS30052M2	Colon cancer, Mesenteric lymph node
38	Lymph node	28	NAT	1'	Proteogenex	014836T4	Surgery
39	Lymph node	47	NAT	1'	Proteogenex	08578T4	Surgery
40	Lymph node	31	NAT	1'	Proteogenex	131254T4	Surgery
41	Muscle	44	NAT		Cleveland Clinic	208C166Ca	Surgery
42	Muscle	49	NAT		Cleveland Clinic	210C106Cb	Surgery
43	Muscle	34	PM	2'	ILSBIO	ILS2186D01	MVA
44	Muscle	45	PM	2'	ILSBIO	ILS1978B02-DS3	MVA
45	Muscle	30	PM	2'	ILSBIO	ILS2103G01-DS4	MVA
46	Pancrea s	29	NAT		Cornell	01-0154 4-2	Surgery
47	Pancrea s	38	NAT		U of Michigan	05-0759	Surgery
48	Pancrea s	42	NAT		U of Michigan	05-1072	Surgery
49	Pancrea s	48	NAT		U of Michigan	05-0420	Surgery
50	Pancrea s	25	NAT		U of Michigan	05-0040	Surgery
51	Skin	48	NAT		Cornell	01-0413 4-2	Surgery
52	Skin	26	PM	45"	ILSBIO	ILS11102C04	MVA
53	Skin	45	PM	2'30"	ILSBIO	ILS11301C01	MVA
54	Skin	28	NAT		U of Michigan	04-0344	Surgery
55	Skin	38	NAT		U of Michigan	08-1337	Surgery
56	Small Bowel	25	NAT		Cleveland Clinic	202C052Ca	Surgery
57	Small Bowel	44	NAT	1'	Proteogenex	32140T1	Surgery
58	Small Bowel	44	NAT		U of Michigan	06-0676	Surgery
59	Small Bowel	30	NAT		U of Michigan	07-1047	Surgery
60	Small Bowel	49	NAT		U of Michigan	04-0274	Surgery
61	Spleen	29	NAT		Cornell	01-0154 5-2	Surgery
62	Spleen	47	NAT		U of Michigan	04-0434	Surgery
63	Spleen	38	NAT		U of Michigan	05-0759	Surgery
64	Spleen	34	NAT		U of Michigan	06-0309	Surgery
65	Spleen	40	NAT		U of Michigan	07-0318	Surgery
66	Stomach	46	NAT		Asterand	1177023F	Surgery
67	Stomach	44	NAT	5"	ILSBIO	USA00341N03	Stomach cancer
68	Stomach	38	NAT	5"	ILSBIO	AGR00246N02	Stomach cancer
69	Stomach	47	Adjacent normal		U of Michigan	07-0565	
70	Stomach	45	Adjacent normal		U of Michigan	09-1541	

PM: Postmortem, whole tissue morphology normal

PM; adjacent normal: Postmortem, normal tissue collected adjacent to abnormal area

MAV: Motor Vehicle Accident

NAT: Normal Adjacent to Tumor
